# Developing and validating the nurse-patient relationship scale (NPRS) in China

**DOI:** 10.1186/s12912-024-01941-w

**Published:** 2024-04-22

**Authors:** Yajie Feng, Chaojie Liu, Siyi Tao, Chen Wang, Huanyu Zhang, Xinru Liu, Zhaoyue Liu, Wei Liu, Juan Zhao, Dandan Zou, Zhixin Liu, Junping Liu, Nan Wang, Lin Wu, Qunhong Wu, Yanhua Hao, Weilan Xu, Libo Liang

**Affiliations:** 1 School of Health Administration, Harbin Medical University, Harbin, China; 2 Qiqihar Medical College, Qiqihar, China; 3 Department of Public Health, School of Psychology and Public Health, La Trobe University, 3086 Melbourne, VIC Australia; 4 Department of Health Policy and Management, School of Public Health, Peking University, 100191 Beijing, China; 5 Southwest Hospital, Third Military Medical University (Army Medical University, 400000 Chongqing, China; 6 Xinqiao Hospital, Third Military Medical University (Army Medical University, 400037 Chongqing, China; 7 Jin Shan Hospital of Fudan University, 201508 Shanghai, China; 8 Anhui Medical University, No.1166, Wangjiang West Road, Shushan District, Hefei, Anhui China

**Keywords:** Nurse-patient relationship, Scale, Validity, Reliability

## Abstract

**Background:**

Poor nurse-patient relationship poses an obstacle to care delivery, jeopardizing patient experience and patient care outcomes. Measuring nurse-patient relationship is challenging given its multi-dimensional nature and a lack of well-established scales.

**Purpose:**

This study aimed to develop a multi-dimensional scale measuring nurse-patient relationship in China.

**Methods:**

A preliminary scale was constructed based on the existing literature and Delphi consultations with 12 nursing experts. The face validity of the scale was tested through a survey of 45 clinical nurses. This was followed by a validation study on 620 clinical nurses. Cronbach’s α, content validity and known-group validity of the scale were assessed. The study sample was further divided into two for Exploratory Factor Analysis (EFA) and Confirmatory Factor Analysis (CFA), respectively, to assess the construct validity of the scale.

**Results:**

The Nurse-Patient Relationship Scale (NPRS) containing 23 items was developed and validated, measuring five dimensions: nursing behavior, nurse understanding and respect for patient, patient misunderstanding and mistrust in nurse, communication with patient, and interaction with patient. The Cronbach’s α of the NPRS ranged from 0.725 to 0.932, indicating high internal consistency. The CFA showed excellent fitness of data into the five-factor structure: χ^2^/df = 2.431, GFI = 0.933, TLI = 0.923, CFI = 0.939, IFI = 0.923, RMSEA = 0.070. Good content and construct validity are demonstrated through expert consensus and psychometric tests.

**Conclusion:**

The NPRS is a valid tool measuring nurse-patient relationship in China.

**Supplementary Information:**

The online version contains supplementary material available at 10.1186/s12912-024-01941-w.

## Introduction

At present, nurse-patient disputes are common, and a large number of reports focus on the relationship and conflicts between nurses and patients. Despite efforts to alleviate the strained relationship between nurses and patients, it still persists [1]. Patients are usually considered as a passive subject [2, 3]. Research points out that many patients, or most of them, are not able to engage in care for themselves through effective interactions with health workers [4]. Henderson [5] noted that professional domination over patient care causes depersonalization and, consequently, worsening of the relationship between the nurse and the patient [2, 6].

A positive nurse-patient relationship is fundamental for effective and high-quality nursing care. The importance of defining and evaluating the connotation of the nurse-patient relationship has been well-established, with a variety of theories being proposed [7–9]. Some scholars define it as a kind of interpersonal relationship in the process of providing and receiving nursing services. Nurses and patients learn and encourage each other, naturally forming a relationship of helping and being helped [10]. Others see it as instrumental, primarily reflecting the help nurses provide to patients [11]. From the perspective of nurses, a positive nurse-patient relationship allows them to effectively plan, provide, and evaluate nursing services. For patients, the caring consciousness, wisdom, and interpersonal skills of nurses are essential for developing and maintaining a continuous nurse-patient relationship [12]. Clinical and interpersonal skills are the two equally important pillars of patient-centered nursing practice [13].

It is critical for nurses to form a positive attitude towards patients that involves respect, trust, and understanding to enable effectively communication and delivery of the help and guidance needed by the patients [14]. Empirical evidence suggests that the tension between nurses and patients is associated with a lack of respect and understanding of nursing care from patients. Some patients or the public may hold inherent prejudices toward the status and nature of nursing work, resulting in a lack of respect and understanding for nurses [15]. This can manifest in behaviors such as not treating nurses with respect or understanding their role. In some extreme cases, patients may resort to verbal and even physical violence against nurses, which can have a negative impact on the nurse-patient relationship. As a result, the nurses may be unable to provide high-quality nursing services [16].

A reliable tool measuring nurse-patient relationship can not only help to better understand the nursing care process, but also predict patient experience and care outcomes [7–9]. However, the existing validated tools measuring the nurse-patient relationship have several limitations. Firstly, there is a lack of comprehensiveness, with most focusing on specific selected aspects of the nurse-patient relationship, such as trust [17, 18], social interaction [19], and care behavior [20]. Secondly, there exists ambiguity in the conceptualization of the elements measured by the existing tools: for example, “respect” can be regarded as an attribute of trust [21] or nursing behavior [20, 22]. Thirdly, the existing tools have failed to consider the special circumstances of nursing work environments in China. The hierarchical and collectivist culture in China has significant implications for how nurses work with their patients and colleagues. Nurses often become an easy target for patient complaints although system problems are usually the underlying reasons [23]. Therefore, there is a need to develop a measurement tool that can capture the complex nature of nurse-patient relationship, especially under the context of the Chinese health system [24].

This study aimed to address the gap in the literature by developing and validating a scale that measures the nurse-patient relationship comprehensively from the perspective of nurses in China, guided by existing theories and considering the existing measurement tools.

## Methods

**Fig. 1:**
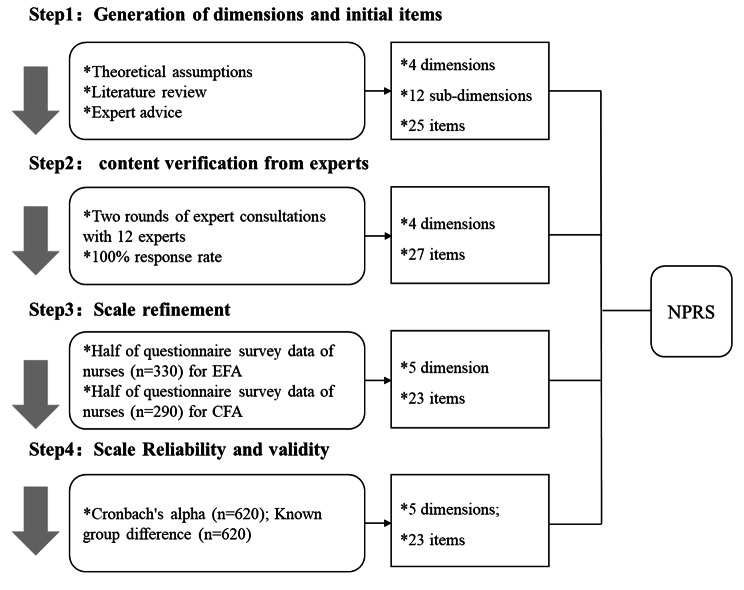
Four steps in scale development. (Note: EFA– Exploratory Factor Analysis; CFA– Confirmatory Factor Analysis; NPRS– Nurse-Patient Relationship Scale)

The study followed the best practice in scale development [25], which involved four steps: item generation, content verification, scale refinement, and reliability and validity assessment (Fig. 1).

The study was conducted in Heilongjiang, a province with a socioeconomic development index at the lower end range in China. In 2019, Heilongjiang had 26 nurses per 10,000 population, compared with a national average of 32 [26].

### Item generation

The concept of nurse-patient relationship was defined as a therapeutic relationship in line with Peplau’s interpersonal relationship theory. Nurses play a variety of roles in helping patients, ranging from a communicator to a caregiver [12]. At the core of the relationship is trust, communication, mutual understanding, and clinical care. Halldorsdottir (2008) likened the two extremes of nurse-patient relationship as “bridge” and “wall” [27]. “Bridge” symbolizes openness of communication and connectivity felt by patients in their relationship with nurses. It represents patient-centeredness and easy access to nursing services. By contrast, “wall” symbolizes a lack of communication and indifference of nurses to patient demands, as well as mistrust between the two parties [27]. The items generated in this study covered both “wall” and “bridge” aspects in relation to trust, communication, understanding, and clinical care.

The sources of items came from a cascading decomposition of the aforementioned theoretical assumptions, a review of the existing measurement tools, and descriptive adaptation to the local health system and clinical practices. A total of 12 sub-domains were mapped into the four core functions of nurse-patient relationship through the process, with advisory support from six external experts who had complementary knowledge and expertise to the research team (Table 1).

**Table 1:** Conceptual framework guiding the development of the nurse-patient relationship scale

Core function	Sub-domain
Mutual understanding	Understanding of patient needs Respect to patients Patient understanding and respect to nurses
Trust	Nurse trust in patients Patient trust in nurses
Communication	Communication plan and preparation Communication process Patient feedback on communication Patient accessibility to nurses when needed
Clinical care	Timeliness of care Quality of care Patient care outcome

### Content verification - Delphi consultations

The Delphi method is one of the most commonly used procedures to establish content validity of a scale [28]. In this study, eligible participants of the Delphi consultations were the experts with a background of nursing research, clinical nursing, or psychology. A minimal of ten years of work experience in the relevant areas was required. The participants were recruited through a stratified convenience sampling strategy. In total, 12 experts from eight provinces participated in the Delphi consultations, covering the eastern developed, the central developing, and the western under-developed regions in China. Half of them worked in academic institutions and half in the healthcare industry.

The participants were invited to respond to the consultation questionnaire by email in December 2019. They were asked to rate the relative importance of each sub-domain on a five-point Likert scale ranging from 1 (disagree) to 5 (agree), and the relevance of each item to its respective sub-domain on a five-point Likert scale ranging from 1 (not relevant) to 5 (essential). Suggestions about modification, removal, or addition of items, sub-domains, and domains were also encouraged. Participation in the consultations was voluntary and verbal informed consent was obtained from each participant.

Consensus of the expert ratings was indicated by the percentage of agreement. The items/sub-domains that had a higher than 80% expert agreement and an over 4 average score were retained [29]. Two rounds of consultations were conducted. The first round resulted in some changes in the subdomains and items, although the four core functions (domains) remained unchanged. In round two, feedback of the round one results was provided, which included the rating results and the corresponding changes made such as removal, addition, and modification of items, sub-domains, and domains. Participants were asked to reconsider their ratings if needed. The 12 experts completed both rounds of consultations.

We also calculated the item content validity index (I-CVI) and the scale content validity index (S-CVI)/average: I-CVI > 0.78 and (S-CVI)/average of 0.90 or higher were deemed acceptable [30, 31].

### Pilot testing

The NPRS endorsed by the experts was tested in a convenience sample of 45 nurses selected from the clinical units (mainly internal medicine, surgery, ICU, and stomatology) of a tertiary hospital in Harbin, capital of Heilongjiang province. Participants were asked to self-complete the paper questionnaire independently. Cronbach’s α coefficient of the scale reached 0.795. No further changes were made as a result of the pilot testing.

### Reliability and validity assessment

Reliability and validity of the NPRS were assessed through a questionnaire survey of clinical nurses in a public tertiary hospital in Qiqihar city in Heilongjiang province. The hospital employed 1093 clinical nurses who had direct contacts with patients. From 29 to 31 December 2019, the nurses working in the clinical units were invited to participate in the survey. Participation in the survey was anonymous and voluntary. Return of the questionnaire was deemed informed consent. In total, 721 questionnaires were distributed and 708 (86.5%) were returned. After removal of the invalid returned questionnaires, 620 (86.0%) were included for data analysis, representing 56.7% of the entire nursing workforce in the participating hospital.

### Ethical considerations

Ethics approval for the study protocol was granted by the research ethics committee of Harbin Medical University.

### Data analysis

Data were analyzed using SPSS 21.0 and AMOS 24.0. A two-sided *p* value of less than 0.05 was considered statistically significant. A pairwise strategy was adopted in managing missing values.

Each item of the NPRS was rated on a five-point Likert scale, ranging from 1 (Strongly disagree) to 5 (Strongly agree). The direction of item scores was aligned before a summed score was calculated for each domain and the entire scale, with a higher score indicating a more positive nurse-patient relationship.

Construct validity was tested through exploratory factor analysis (EFA) and confirmatory factor analysis (CFA). The study sample was randomly divided into two mutually independent sub-samples, with 330 participants for EFA and 290 participants for CFA, respectively. The appropriateness of factor analyses was assessed using the Kaiser-Meyer-Olkin (KMO) measure (KMO ≥ 0.50) and Bartlett’s test of sphericity (*p* < 0.05) [32]. The EFA extracted factors with an eigenvalue greater than 1 using principal component analysis (PCA) with maximal rotation of variance. This allowed us to identify and eliminate poorly-fitted items, including those with a low factor load (< 0.4) on all factors and those with a high load (≥ 0.4) across multiple factors [33]. The CFA then assessed the fitness of data into the adjusted scale resulting from the EFA. A good model fit was indicated by Chi-square/degree of freedom (χ^2^/df ratio ranging from 1 to 3), goodness-of-fit index (GFI > 0.9), root mean square error of approximation (RMSEA < 0.08), a root mean square residual (RMR < 0.08), a comparative fit index (CFI > 0.9), a normalized fit index (NFI > 0.9), and Incremental Fit Index (IFI > 0.9) [34]. Convergent validity was assessed by composite reliability (CR > 0.70) [35] and average variance extracted (AVE > 0.5) from CFA [36]. Discriminant validity was assessed by comparing AVE with the Pearson correlation coefficients between domains: A good discriminant validity is indicated if the square root of AVE of each construct is greater than its correlations with the rest of the constructs [37, 38].

Reliability was assessed by Cronbach’s α for the entire NPRS and its domains using the entire sample. A greater than 0.7 Cronbach’s α coefficient indicates good internal consistency [39].

Known-group validity was tested through student *t* tests using the entire sample, with a hypothesis that nurse-patient relationship varies by the personal characteristics of the nurse [40, 41].

## Results

### Content validity

#### Characteristics of Delphi participants

About one third of the participants of the Delphi consultations came from Heilongjiang province and over 40% aged between 30 and 40 years. Half held a doctoral degree and had more than 20 years of work experience. Over 58% of participants held a senior professional title (Table 2).

**Table 2:** Characteristics of Delphi participants (*n* = 12)

Characteristics		N	%
Region	Heilongjiang	4	33.3
	Zhejiang	1	8.3
	Hubei	2	16.7
	Shanghai	1	8.3
	Beijing	1	8.3
	Hunan	1	8.3
	Ningxia	1	8.3
	Shandong	1	8.3
Age (Years)	30–40	5	41.7
	41–50	4	33.3
	> 50	3	25.0
Work experience (Years)	10–20	6	50.0
	21–30	1	8.3
	> 31	3	25.0
Highest qualification	Doctoral degree	6	50.0
	Master’s degree	3	25.0
	Undergraduate degree	3	25.0
Professional title	Senior	7	58.3
	Associate senior	3	25.0
	Intermediate	2	16.7

#### Results of Delphi consultations

The first round of consultations resulted in an increase of items from 25 to 27: five new items were suggested while three were removed (Table 3). The three items that were suggested by some experts for removal all had low levels of expert agreement. Wording changes were also suggested by the experts for nine items to reduce ambiguity and improve clarity (Supplementary Table 1). The four core functions (domains) remained unchanged.

**Table 3:** Results of expert consultations (*n* = 12)

Item	First Round				Item	Second Round		
	Source	Description	Agreement* % (Mean)	Suggestion		Modified description	Agreement* % (Mean)	Suggestion
**Dimension 1: nurse-patient understanding and respect**								
1	Peplau's relationship theory [42]	I understand what it's like to be sick	66.6% (3.75)	Adjust expression	N1	I can understand and respect the feelings of patients when they are sick	100% (4.69)	Finetune expression
2	Caring Behavior Assessment [43]	I can't call the patient kindly	83.3% (4.75)	Adjust expression	N2	I can call the patient kindly	100% (4.67)	
3	Caring Behavior Assessment [43]	I don't like to spend time listening to patients express concerns about their illness	83.3% (4.08)	Adjust expression	N3	I'm not willing to spend time listening to patients' concerns about their condition	100% (4.58)	
4	Caring Behavior Assessment [43]	I can protect the patient's information and privacy	100% (4.75)		N4	I can protect the patient's information and privacy	100% (4.83)	
5	Caring Behavior Assessment [43]	I have no patience for patients with poor expression skills	100% (4.08)	Adjust expression	N5	I am also patient with patients who cannot describe the disease in detail	100% (4.58)	
6	Caring Behavior Assessment [43]	I'm not prejudiced against the patients I administer	83.3% (4.17)		N6	I have no prejudice against the patients I care for	100% (4.67)	
7	Caring Behavior Assessment [43]	Patients show bias and discrimination against my work	75.0% (4.00)	Adjust expression	N7	Patients show bias and discrimination against the nature of my work	100% (4.58)	
8	Caring Behavior Assessment [43]	The patient's address to me is rude	75.0% (4.08)	Adjust expression	N8	The patient is very rude to me	91.6% (4.50)	
9	Caring Behavior Assessment [43]	Patients don't cooperate with my work	41.7% (3.83)	Delete				
**Dimension 2 nurse-patient trust**								
10	Okaya Keiko Trust Scale [44]	I don't trust the Information provided by the patient	75.0% (3.92)	Add more details	N9	I don't trust the Information provided by the patient	83.3% (3.92)	
11	Okaya Keiko Trust Scale [44]	I’m on guard against patients	75.0% (3.75)	Delete				
12	Okaya Keiko trust Scale [44]	I’m afraid the patient is a threat to my personal safety	41.7% (3.67)	Retained because item 11 was removed		I’m afraid the patient is a threat to my personal safety	66.7% (4.00)	Delete
13	Okaya Keiko Trust Scale [44]	Patients have questioned the performance of my nursing practices	75.0% (4.17)	Add more details	N10	patients have questioned the performance of my nursing operations and professional skills	91.6% (4.50)	
14	Okaya Keiko Trust Scale [44]	Patients or family members often supervise me when administering medication	83.3% (4.08)	Adjust expression	N11	When caring for a patient, the patient or the patient’s family often supervises me	83.3% (4.17)	
		Patients do not trust my explanation and health education		Add	N12	patients do not trust my explanation and health education	91.6% (4.33)	
**Dimension 3 nurse-patient communication**								
15	Nurse-patient Communication Questionnaire	I don't have enough energy to answer questions from patients or their families	83.3% (4.25)	Add more details	N13	I do not have enough energy to patiently answer questions from patients or their families	100% (4.58)	
16	Nurse-patient Communication Questionnaire	I think a lot of what the patient says is useless, so it is unlikely that I will interrupt him/her quickly	75.0% (4.00)		N14	I think a lot of the patient's words are useless, so I will interrupt him / her soon	91.6% (4.50)	
17	Nurse-patient Communication Questionnaire	I think my words are easy to understand and I don't need to spend time explaining them to the patient	66.7% (4.08)	Adjust expression	N15	I think I have clearly expressed my meaning and I don’t need to spend time explaining to patients	91.6% (4.42)	
18	Nurse-patient Communication Questionnaire	I will not voluntarily apologize to patients for my failures in care	66.7% (4.00)	Merge of item 18 and 19	N16	I will voluntarily apologize to patients for my failures in care	100% (4.33)	Adjust expression
19	Communication Questionnaire	Patients often overreact during communication attitude	75.0% (4.08)	Merge of item 18 and 19				
20	Communication Questionnaire	In the process of communication, the patient's family members often speak excessively	75.0% (4.00)	Adjust expression	N17	During the communication process, the patient or the patient's family often express excessive emotion	91.6% (4.50)	
		Before special examination or surgery, I can inform the patient of the matters needing attention in time		Add	N18	Before special examination or surgery, I can inform the patient of the matters needing attention in time	100% (4.67)	
		Maintain proper eye contact when communicating with patients		Add this item according to expert opinions	N19	Maintain proper eye contact when communicating with patients	91.6% (4.58)	
		patient or family member will thank me for the care operation		Add this item according to expert opinions	N20	patient or family member will thank me for the care operation	100% (4.67)	
**Dimension 4 nurses' help and guidance to patients**								
21	Caring Behavior Assessment [43]	I encourage patients to call me when they have problems	100% (4.83)		N21	I encourage patients to call me when they have problems	100% (4.92)	
22	Caring Behavior Assessment [43]	I can give patients routine nursing operations in a timely manner	100% (4.75)		N22	I can give patients routine nursing operations in a timely manner	100% (4.92)	
23	Humanistic Nurse-Patient Scale [45]	When a patient has an emergency, I can correctly judge and deal with it according to the nursing standard	100% (4.67)		N23	When a patient has an emergency, I can correctly judge and deal with it according to the nursing standard	100% (4.83)	
24	Humanistic Nurse-Patient Scale [45]	I have enough time to give patients the appropriate guidance and health education	100% (4.67)	Add more details	N24	I have enough time and ability to give patients corresponding guidance and health education	91.6% (4.75)	
25		I can relieve the patient's symptoms	75.0% (4.17)	Add more details	N25	I can relieve the pain and stress of patients through my nursing work	100% (4.67)	
		I can basically solve the patient's nursing problems		Add	N26	I can basically solve the patient's nursing problems	91.6% (4.50)	

Note: *including both “agree” or “strongly agree”

The first round of Delphi consultations already achieved an I-CVI of 0.83 (22/25) and an (S-CVI)/average of 0.98, exceeding the recommended value.

The second round of consultations led to language modification of two items. One item was removed because it failed to reach agreement among the experts in both rounds of consultations (Table 2). This resulted in a final version of the NPRS, containing 26 items, measuring nurse patient understanding and respect (8 items), nurse-patient trust (4 items), nurse-patient communication (8 items), and nurse’s help and guidance to patients (6 items). The second round of Delphi consultations already achieved an I-CVI of 0.83 (22/26) and an (S-CVI)/average of 0.99, exceeding the recommended value.

### Construct validity

#### Characteristics of survey participants

Of the 620 clinical nurses surveyed, 88.1% were female and 46.0% aged between 26 and 35 years. Most were married (53.2%), obtained a university degree (59.0%), and worked in internal medicine (55.6%). Almost half (49.0%) had over five years of work experience and 70.6% held an intermediate or senior professional title. The two sub-divided samples had slightly different characteristics of study participants (Table 4).

**Table 4:** Sociodemographic characteristics of study participants

Variables	Total (n = 620)	Sample One (n = 330)	Sample Two (n = 290)	*p*
	N (%)	N (%)	N (%)	
**Gender**				0.041*
Male	27 (4.4)	13 (3.9)	14 (4.8)	
Female	546 (88.1)	293 (88.8)	253 (87.2)	
Other	47 (7.6)	34 (7.3)	23 (7.9)	
**Age (Years)**				0.000***
18 ~ 25	211 (34.0)	123 (37.3)	88 (30.3)	
26 ~ 35	285 (46.0)	144 (43.6)	141 (48.6)	
36 ~ 52	111 (18.0)	57 (17.3)	58 (20.0)	
Missing	13 (2.0)	6 (0.02)	3 (1.0)	
**Educational attainment**				0.010**
College / High School	239 (38.5)	131 (39.7)	108 (37.2)	
Bachelor’s degree and above	366 (59.0)	189 (57.3)	177 (60.9)	
Missing	15 (2.4)	15 (2.4)	5 (1.7)	
**Work experience (Years)**				0.003**
≤5	297 (47.9)	161 (48.8)	136 (46.9)	
6–10	166 (26.8)	85 (25.6)	81 (27.9)	
≥11	138 (22.3)	70 (21.2)	68 (23.4)	
Missing	19 (3.0)	14 (0.04)	5 (1.7)	
**Only child in family**				0.481
Yes	446 (71.9)	246 (74.5)	200 (69.0)	
No	153 (24.7)	72 (21.8)	81 (27.9)	
Missing	21 (3.4)	12 (3.7)	9 (3.1)	
**Work department**				0.079
Internal medicine	358 (57.7)	198 (60.0)	160 (55.2)	
Surgical, Obstetrics and Gynecology	224 (36.1)	110 (33.3)	114 (39.3)	
Missing	38 (7.2)	22 (6.7)	16 (5.5)	
**Professional title**				0.000***
Junior/No title	486 (78.4)	265 (80.3)	221 (76.2)	
Intermediate title and above	123 (19.8)	58 (17.5)	65 (22.3)	
Missing	11 (0.02)	6 (1.8)	4 (1.4)	
**Marital status**				0.007**
Unmarried	280 (45.2)	158 (47.9)	122 (42.1)	
Married	330 (53.2)	165 (50.0)	165 (56.9)	
Other	10 (1.6)	7 (2.1)	3 (1.0)	

Note: * *p* < 0.05; ** *p* < 0.01, ***p < 0.001

#### Structural adjustment of the scale

The KMO (0.903) and Bartlett test of sphericity (*p* < 0.001) indicated appropriateness of the subsample (*n* = 330) for EFA. The EFA extracted five factors: *nursing behavior*; *nurse understanding and respect for patient; patient misunderstanding and mistrust; communication with patient; and interaction with patient.* The five factors explained 68.06% of the total variance. Three items (item N7, N9, N16) with low factor loadings or cross loadings were removed, resulting in a 23-item NPRS (Table 5). The complete NPRS scale is shown in supplementary Table S3.

**Table 5:** Results of exploratory factor analysis (*n* = 330)

Item	Factor				
	1	2	3	4	5
**Nursing behavior**					
I encourage patients to call me when they have problems (N21)	**0.827**				
I can give patients routine nursing operations in a timely manner (N22)	**0.855**				
When a patient has an emergency, I can correctly judge and deal with it according to the nursing standard (N23)	**0.880**				
I have enough time and ability to give patients corresponding guidance and health education (N24)	**0.816**				
I can relieve the pain and stress of patients through my nursing work (N25)	**0.804**				
I can basically solve the patient’s nursing problems (N26)	**0.835**				
**Nurse understanding and respect for patient**					
I can understand and respect the feelings of patients when they are sick (N1)		**0.790**			
I can call the patient affectionately (N2)		**0.819**			
I have no prejudice against the patients I care for (N3)		**0.795**			
I can protect the patient’s information and privacy (N4)		**0.829**			
I am also patient with patients who cannot describe the disease in detail (N5)		**0.711**			
**Patient misunderstanding and mistrust in nurse**					
The patient is very rude to me (N8)			**0.655**		
Patients have questioned the performance of my nursing operations and professional skills (N10)			**0.785**		
When caring for a patient, the patient or the patient’s family often supervises me (N11)			**0.845**		
Patients do not trust my explanation and health education (N12)			**0.842**		
During the communication process, the patient or the patient’s family often express excessive emotion (N17)			**0.627**		
**Communication with patient**					
I’m not willing to spend time listening to patients’ concerns about their condition (N6)				**0.674**	
I do not have enough energy to patiently answer questions from patients or their families (N13)				**0.752**	
I think a lot of the patient’s words are useless, so I will interrupt him/her soon (N14)				**0.795**	
I think I have clearly expressed my meaning and I don’t need to spend time explaining to patients (N15)				**0.766**	
**Interaction with patient**					
Before special examination or surgery, I can inform the patient of the matters needing attention in time (N18)					**0.592**
Maintain proper eye contact when communicating with patients (N19)					**0.653**
Patient or family member will thank me for the care operation (N20)					**0.595**
Eigen value	5.92	3.96	3.78	2.44	1.59
Explained variance (%)	22.77	15.22	14.54	9.40	6.13
Cumulative variance (%)	22.77	38.00	52.54	61.94	68.06

#### Construct validity

The KMO (0.902) and Bartlett test of sphericity (*p* < 0.001) indicated appropriateness of the subsample (*n* = 290) for CFA. Excellent fitness of data into the five-factor structure in line with the EFA was found: χ^2^/df = 2.431, GFI = 0.933, TLI = 0.923, CFI = 0.939, IFI = 0.923, and RMSEA = 0.070. The vast majority of items had a factor loading greater than 0.70 on its respective domain (Supplementary Table S2).

#### Convergent and discriminatory validity

Convergent validity of the scale was confirmed by the CFA (*n* = 290), as indicated by the greater than 0.7 CR and greater than 0.5 AVE (Table 6).

The five domains were moderately correlated. The square root of the AVE value of each domain generated from the CFA (*n* = 290) was much greater than its correlation coefficients with other domains (Table 6), indicating good discriminant validity between dimensions.

**Table 6:** Composite reliability and discriminant validity of the scale (*N* = 290)

Domain	No. of items	Composite reliability	Correlation coefficients (Square root of average variance extracted)				
			1	2	3	4	5
Nursing behavior	6	0.926	(0.823)				
Nurse understanding and respect for patient	5	0.918	0.209^**^	(0.831)			
Patient misunderstanding and mistrust in nurse	5	0.879	0.172^**^	0.127^**^	(0.771)		
Communication with patient	4	0.829	0.264^**^	0.178^**^	0.333^**^	(0.743)	
Interaction with patient	3	0.905	0.297^**^	0.221^**^	-0.097^*^	-0.228^**^	(0.873)

Note: * *p* < 0.05; ** *p* < 0.01

#### Cronbach’s α

High levels of internal consistency were found for the entire scale and its five domains, as indicated by the higher than 0.7 Cronbach’α coefficients (Table 7).

**Table 7:** Cronbach’s α coefficients of the scale (*n* = 620)

Domain	Number of items	Mean ± SD	Cronbach’s α
Nursing behavior	6	24.70 ± 3.77	0.932
Nurse understanding and respect for patient	5	20.46 ± 3.36	0.903
Patient misunderstanding and mistrust in nurse	5	22.90 ± 5.79	0.819
Communication with patient	4	15.58 ± 3.04	0.787
Interaction with patient	3	12.13 ± 2.29	0.865
Total	23	95.77 ± 13.41	0.725

Note: SD - standard deviation

#### Known group validity

There were statistically significant differences in the NPRS scores by gender and working experience (Table 8). Male nurses had lower scores (indicating poorer relationship) in two domains: patient misunderstanding and mistrust in nurse, and communication with patients, compared to female nurses (*p* < 0.01). Longer work experience was associated with higher scores (indicating better relationship) in two domains: nurse understanding and respect for patients, and interaction with patients (*p* < 0.05). Patient complaint was associated with a lower score (indicating poorer relationship) in one domain (patient misunderstanding and mistrust in nurse) despite a lack of significance in the difference of overall NPRS scores.

**Table 8:** Scale scores of participants with different characteristics (*n* = 620)

Variable	N	NPRS	Nurse behavior	Nurse understanding and respect for patient	Patient misunderstanding and mistrust in nurse	Communication with patient	Interaction with patient
***Gender***							
Male	27	82.30 ± 10.89*	20.74 ± 3.04	20.67 ± 2.96	14.22 ± 5.05***	13.93 ± 3.95***	12.74 ± 1.97
Female	546	85.18 ± 11.62	20.72 ± 2.82	20.39 ± 3.36	16.23 ± 4.37	15.73 ± 2.93	12.11 ± 2.27
***Age (Years)***							
≤25	211	85.75 ± 12.59	20.78 ± 3.45	20.40 ± 3.75	16.31 ± 4.78	15.94 ± 3.27	12.41 ± 2.22
>25	400	84.76 ± 11.44	20.71 ± 2.97	20.53 ± 3.14	16.04 ± 4.23	15.49 ± 2.92**	12.00 ± 2.34
***Work experience (Years)***							
≤5	297	84.56 ± 12.67	20.62 ± 3.49	20.16 ± 3.81	16.16 ± 4.61	15.52 ± 3.27	12.10 ± 2.42
>5	304	85.49 ± 10.92*	20.84 ± 2.76	20.75 ± 2.83**	16.08 ± 4.32	15.65 ± 2.79	12.18 ± 2.18**
***Qualification***							
College / High School	239	85.18 ± 12.43	20.55 ± 3.51	20.47 ± 3.21	16.36 ± 4.46	15.62 ± 3.24	12.18 ± 2.45
Bachelor degree or higher	366	84.93 ± 11.42	20.80 ± 2.86	20.45 ± 3.46	16.02 ± 4.44	15.58 ± 2.91	12.08 ± 2.18
***Marital status***							
Married	330	85.27 ± 11.31	20.77 ± 2.76	20.68 ± 2.96	16.15 ± 4.35	5.64 ± 2.90	12.03 ± 2.25
Not in a marriage	287	84.72 ± 12.41	20.62 ± 3.53	20.21 ± 3.75	16.10 ± 4.53	15.56 ± 3.18	12.24 ± 3.33
***Patient complaint***							
Yes	91	82.60 ± 10.80	20.65 ± 3.38	20.33 ± 2.73	14.54 ± 4.14**	15.04 ± 2.79	12.04 ± 2.29
No	522	85.38 ± 11.81	20.71 ± 3.02	20.47 ± 3.44	16.39 ± 4.40	15.68 ± 3.07	12.14 ± 0.2.26
***Only child in family***							
Yes	446	85.08 ± 11.95	20.74 ± 3.13	20.42 ± 3.42	16.19 ± 4.58	15.62 ± 3.11	12.11 ± 2.38
No	153	84.56 ± 11.01	20.61 ± 3.11	20.50 ± 3.12	15.88 ± 3.85	15.40 ± 2.83	12.17 ± 2.04
***Work department***							
Internal medicine	358	84.31 ± 11.88	20.70 ± 3.10	20.09 ± 3.52	15.85 ± 4.27	15.47 ± 3.03	12.20 ± 2.18
Surgical, Obstetrics and Gynecology	224	85.83 ± 11.87	20.70 ± 3.29	20.81 ± 2.87**	16.33 ± 4.66	15.90 ± 2.99	12.09 ± 2.48
***Professional title***							
Junior/No title	486	85.20 ± 12.07	20.75 ± 3.23	20.37 ± 3.41	16.23 ± 4.44	15.67 ± 2.06	12.17 ± 2.30
Intermediate title and above	123	84.11 ± 10.47	20.45 ± 2.49	20.71 ± 3.20	15.68 ± 4.20	15.41 ± 2.66	11.86 ± 2.25

Note: * *p* < 0.1; ** *p* < 0.05; *** *p* < 0.01

## Discussion and conclusions

### Discussion

The current research represents an attempt to provide a clear conceptualization and a reliable and valid scale measuring the comprehensive nurse-patient relationship in China. This research closely followed the best practice in scale development, involving a series studies covering the generation of dimensions and initial items, verification of the content, refinement of the scale, and reliability and validity testing of the scale. Previous studies have endeavored to assess the nurse-patient relationship through specific theories [18, 46, 47]. The nurse-patient relationship is indeed multifaceted. From a practical standpoint, no single theory can entirely encapsulate the nature of the nurse-patient relationship. The nurse-patient relationship scale developed in this current study offers a comprehensive tool by incorporating and refining dimensions and items derived from previous studies.

The results showed that the NPRS developed by our research has good reliability and validity. It supports a multi-dimensional construct, with Cronbach’s alpha of the scale and its five domains well exceeding the acceptable value of 0.7. Good content and construct validity are demonstrated through expert consensus and psychometric tests.

The NPRS has captured all of the essential elements of nurse-patient relationship as measured by the existing measurement tools, including trust [18, 48], communication and interaction [46, 49–51], and respect and humanistic care [47]. It covers both positive and negative behavioral reflections of the nurse-patient relationship, and puts nursing responsiveness, care process, and care outcomes at the core of the relationship. Mutual understanding, trust and respect provide the foundation for a positive nurse-patient relationship [27], which enables positive behaviors and interactions between the two to ensure good care outcomes.

The NPRS can help managers and policymakers to better respond to the call for patient-centered care. Increasing tensions in the relationship between nurses and patients due to various reasons have been observed worldwide [52], prompting calls for improving work and cultural environments. In this current study, we found that patient complaints are associated with poorer nurse-patient relationship, characterized by patient misunderstanding and distrust in nurses. Indeed, experiencing patient complaints reduces job satisfaction and the quality of working life of nurses [53]. Nurses facilitate care through frequent and direct contact with patients and their families in almost all healthcare settings, particularly in hospitals [54]. Patient demands and expectations have never been so high due to the rapid technological advancement and increased affordability of care [55]. What follows is the increase in the workload and the high pressure imposed on nurses [56]. Constant and chronic occupational stress produce burnout, a prominent characteristic of nursing work [57]. Study shows that the inverse relationship between physician burnout and patient safety affects nurse-patient relationship [58]. On the other hand, patients may take improved care outcomes for granted [59]. Therefore, it is important to use a tool, such as the NPRS, to help nurses and their managers to identify key domains in the nurse-patient relationship for improvement.

Our findings have some policy implications on the current health system reform in China. We found that the male nurses have worse relationship with patients than their female counterparts. This may reflect the structural inequality in gender division of work: Female nurses take most of the care tasks [60]. Female nurses may be more sensitive than their male counterparts, have stronger empathy, communication and caring characteristics, and pay more attention to emotional communication [61]. A study on the humanistic care of male nurses showed that male nurses expressed humanistic care differently from female nurses. Female nurses were more inclined to use their unique mother-like image to care for patients, while male nurses mostly used professional behaviors to care for patients [62]. There is a need to address the gender inequality and strengthen the communication competency of male nurses.

In the current study, we found that longer work experience is associated with a better nurse-patient relationship, in terms of nurse understanding and respect for patient and interaction with patient. Benner argues that rich life experience and increased situation awareness can help nurses to better manage nurse-patient relationship [63]. Empirical evidence shows that nursing students can obtain both professional and personal growth, such as a rise in confidence and self-esteem, through accumulated experience in interactions with patients [64]. However, professional and managerial support is equally, if not more, important to enable nurses to excel in managing nurse-patient relationship. As indicated in the findings of this current study, longer work experience does not appear to improve nurse behavior, patient misunderstanding and mistrust in nurse, and communication with patient.

#### Limitation

The current study has some limitations. The study sample was drawn from one hospital. Future studies should expand participants to a more representative sample. It is also important to examine the tool from patient perspective. The NPRS was developed under the context of the Chinese health system. Cross-cultural adaptation is needed should it be used in different health system settings.

### Conclusion

The 23-item NPRS is a valid tool measuring the comprehensive relationship between nurses and patients under the context of the Chinese health system. It measures five domains: nursing behavior, nurse understanding and respect for patient, patient misunderstanding and mistrust in nurse, communication with patient, and interaction with patient. The NPRS presents an opportunity for nurses and their managers to reflect and identify key domains in nurse-patient relationship for improvement. Healthcare practitioners and policymakers can utilize this tool to pinpoint crucial areas for enhancing the development of a trusting and productive nurse-patient relationship.

### Electronic supplementary material

Below is the link to the electronic supplementary material.

Supplementary Material 1

Supplementary Material 2

## Data Availability

The data sets used and/or analyzed during the current study are available from the corresponding author on reasonable request.

## References

[1] Ferri P, Silvestri M, Artoni C (2016). Workplace violence in different settings and among various health professionals in an Italian general hospital: a cross-sectional study[J]. Psychol Res Behav Manage.

[2] Cahill J (1998). Patient participation–a review of the literature[J]. J Clin Nurs.

[3] Truglio-Londrigan M (2015). The patient experience with Shared decision making: a qualitative descriptive Study[J]. J Infus Nurs.

[4] Grffith R, Tengnah C (2013). Shared decision-making: nurses must respect autonomy over paternalism[J]. Br J Community Nurs.

[5] Henderson S (2003). Power imbalance between nurses and patients: a potential inhibitor of partnership in care[J]. J Clin Nurs.

[6] Malaquin-Pavan E. [Is nursing care an intrusion? [J]. Soins, 2015(794):21. 26043627

[7] Feo R, Rasmussen P, Wiechula R (2017). Developing effective and caring nurse-patient relationships[J]. Nurs Standard (Royal Coll Nurs (Great Britain): 1987).

[8] Hagerty BM, Patusky KL (2003). Reconceptualizing the nurse-patient relationship[J]. J Nurs Scholarsh.

[9] Molina-Mula J, Gallo-Estrada J. Impact of nurse-patient relationship on quality of care and patient autonomy in Decision-Making[J]. Int J Environ Res Public Health, 2020,17(3). 10.3390/ijerph17030835 PMC7036952 32013108

[10] Genoveva GG. The nurse-patient relationship as a Caring Relationship[J]. Nurs Sci Q, 2009,22(2). 10.1177/0894318409332789 19342710

[11] Book Review: Nursing Theorists and Their Work (4th ed.) Edited by Ann Marriner Tomey and Martha Raile Alligood (St. Louis, MO: C. V. Mosby. 1998 Reviewed by Burns Nancy, RN; PhD; FAAN Professor, The University of Texas at Arlington[Z]. United States: 1999;12:263.

[12] Interpersonal relations in nursing[J].

[13] Feo R, Rasmussen P, Wiechula R (2017). Developing effective and caring nurse-patient relationships[J]. Nurs Stand.

[14] Dithole KS, Thupayagale-Tshweneagae G, Akpor OA (2017). Communication skills intervention: promoting effective communication between nurses and mechanically ventilated patients[J]. BMC Nurs.

[15] Zhao S, Xie F, Wang J (2018). Prevalence of Workplace Violence against Chinese Nurses and Its Association with Mental Health: a cross-sectional Survey[J]. Arch Psychiatr Nurs.

[16] Bridges J, Nicholson C, Maben J (2013). Capacity for care: meta-ethnography of acute care nurses’ experiences of the nurse-patient relationship[J]. J Adv Nurs.

[17] Radwin LE, Cabral HJ (2010). Trust in nurses Scale: construct validity and internal reliability evaluation[J]. J Adv Nurs.

[18] Ozaras G, Abaan S (2018). Investigation of the trust status of the nurse-patient relationship[J]. Nurs Ethics.

[19] Rask M, Malm D, Kristofferzon ML (2010). Validity and reliability of a Swedish version of the Relationship Assessment Scale (RAS): a pilot study[J]. Can J Cardiovasc Nurs.

[20] Wu Y, Larrabee JH, Putman HP (2006). Caring behaviors Inventory: a reduction of the 42-Item Instrument[J]. Nurs Res.

[21] Zhao Ling W, Rong ZHU, Chenhui (2018). Revision of nurse-patient relationship trust Scale and test of reliability and validity [J]. J Nurs Sci.

[22] He Ting G, Yingying L, Xiaohong (2021). Sinicization and validity test of nursing behavior scale [J]. Shanghai Nurs.

[23] Liu C, Bartram T, Leggat SG. Link of patient care outcome to occupational differences in response to human resource management: a cross-sectional comparative study on hospital doctors and nurses in China[J]. Int J Environ Res Public Health, 2020,17(12). 10.3390/ijerph17124379 PMC7344802 32570912

[24] Feo R, Conroy T, Wiechula R (2019). Instruments measuring behavioural aspects of the nurse–patient relationship: a scoping review[J]. J Clin Nurs.

[25] Boateng GO, Neilands TB, Frongillo EA (2018). Best practices for developing and Validating Scales for Health, Social, and behavioral research: a Primer[J]. Front Public Health.

[26] National Bureau of Statistics. https://data.stats.gov.cn/easyquery.htm?cn=E0103&zb=A0O02&reg=230000&sj=2019.

[27] Halldorsdottir S (2008). The dynamics of the nurse-patient relationship: introduction of a synthesized theory from the patient’s perspective[J]. Scand J Caring Sci.

[28] Hasson F, Keeney S, McKenna H (2000). Research guidelines for the Delphi survey technique[J]. J Adv Nurs.

[29] Bing-Jonsson PC, Bjork IT, Hofoss D (2015). Competence in advanced older people nursing: development of ‘nursing older people–competence evaluation tool‘[J]. Int J Older People Nurs.

[30] Timmins F. Nursing Research Generating and Assessing Evidence for Nursing Practice, Polit DF, Tatano Beck C. Lippincott Williams & Williams, London, ISBN: 978-1-60547-708-4 - scienceD[J]. Nurse Education in Practice. 2012;13(6).

[31] Polit DF, Beck CT (2006). The content validity index: are you sure you know what’s being reported? Critique and recommendations[J]. Res Nurs Health.

[32] Kang H (2013). [A guide on the use of factor analysis in the assessment of construct validity[J]. J Korean Acad Nurs.

[33] WeiLong Z, Shaozhuang M, Li G (2018). Development and validation of doctor-patient relationship scale in China[J]. Health Qual Manage.

[34] Structural Model Evaluation and Modification. An Inverted Estimation Approach[J]. 10.1207/s15327906mbr2502_4 26794479

[35] Wynne WC, Barbara LM, Peter RN. A partial least squares Latent variable modeling approach for measuring interaction effects: results from a Monte Carlo simulation study and an electronic-mail emotion/adoption study[J]. Inform Syst Res, 2003,14(2).

[36] Richard PB. Evaluating structural equation models with unobservable variables and measurement error: a comment[J]. J Mark Res. 1981;18(3).

[37] Fuller CM, Simmering MJ, Atinc G (2016). Common methods variance detection in business research[J]. J Bus Res.

[38] Li C, Jiang Y. Measurement model improvement of organizational memory: the development and validation of local scaling[J]. Res Financial Economic Issues, 2012(10):17–24.

[39] Multivariate. Date Analysis[M].

[40] Moriconi S, Balducci PM, Tortorella A (2020). Aggressive behavior: nurse-patient relationship in Mental Health Setting[J]. Psychiatr Danub.

[41] Boge LA, Dos SC, Moreno-Walton LA (2019). The relationship between Physician/Nurse gender and patients’ correct identification of Health Care Professional roles in the Emergency Department[J]. J Womens Health (Larchmt).

[42] Hildegard P. Interpersonal Relation of Nursing[J]. 1952.

[43] Cronin SN, Harrison B (1988). Importance of Nurse Caring Behaviour as Perceived by patients after myocardial Infarction[J]. Heart Lung J Acute Crit Care.

[44] 岡谷恵子. 看護婦-患者関係における信頼を測定する質問紙の開発-信頼性 妥当性の検定[J]. 1994 年度聖路加看護大学大学院博士論文, 1994.[Z].

[45] Boscart VM, Pringle D, Peter E (2016). Development and psychometric testing of the humanistic nurse-patient Scale[J]. Can J Aging / La Revue Canadienne Du Vieillissement.

[46] Dithole KS, Thupayagale-Tshweneagae G, Akpor OA (2017). Communication skills intervention: promoting effective communication between nurses and mechanically ventilated patients[J]. BMC Nurs.

[47] Boscart VM, Pringle D, Peter E (2016). Development and psychometric testing of the humanistic nurse-patient Scale[J]. Can J Aging.

[48] Burge DM (2009). Relationship between patient trust of nursing staff, postoperative pain, and discharge functional outcomes following a total knee arthroplasty[J]. Orthop Nurs.

[49] Fakhr-Movahedi A, Rahnavard Z, Salsali M (2016). Exploring nurse’s communicative role in nurse-patient relations: a qualitative Study[J]. J Caring Sci.

[50] McGilton KS, Sorin-Peters R, Sidani S (2012). Patient-centred communication intervention study to evaluate nurse-patient interactions in complex continuing care[J]. BMC Geriatr.

[51] Strauss B (2013). The patient perception of the nurse-patient relationship when nurses utilize an electronic health record within a hospital setting[J]. Comput Inf Nurs.

[52] Keykaleh MS, Safarpour H, Yousefian S (2018). The relationship between Nurse’s job stress and patient Safety[J]. Open Access Macedonian J Med Sci.

[53] Teymourzadeh E, Rashidian A, Arab M (2014). Nurses exposure to workplace violence in a large teaching hospital in Iran[J]. Int J Health Policy Manage.

[54] Kieft RA, de Brouwer BB, Francke AL (2014). How nurses and their work environment affect patient experiences of the quality of care: a qualitative study[J]. BMC Health Serv Res.

[55] The power in. the nurse-patient relationship: integrative review[J].

[56] Allande-Cussó R, Macías-Seda J, Porcel-Gálvez AM (2020). Transcultural adaptation into Spanish of the Caring nurse-patient interactions for assessing nurse-patient relationship competence[J]. Enfermería Clínica (English Edition).

[57] Maslach C, Leiter MP. The truth about burnout[M]. The Truth About Burnout; 1997.

[58] Garcia CL, Abreu LC, Ramos J et al. Influence of burnout on patient safety: systematic review and meta-analysis[J]. Med (Kaunas). 2019;55(9). 10.3390/medicina55090553 PMC6780563 31480365

[59] Al-Awamreh K, Suliman M (2019). Patients’ satisfaction with the quality of nursing care in Thalassemia units[J]. Appl Nurs Res.

[60] Gender differences in. sleeping hours and recovery experience among psychiatric nurses in Japan[J].

[61] Zhou Xiaomei L (2019). Investigation and analysis of clinical nurses’ nursing care behavior and patients’ caring perception [J]. Chin J Practical Nurs.

[62] Zhang, Wen (2017). Cheng Xiangwei. A qualitative study on the practice perception of humanistic care in male nurses [J]. J Nurs Sci.

[63] Benner PE. From novice to expert: excellence and power in clinical nursing practice[M]. From novice to expert: excellence and power in clinical nursing practice, 1984.

[64] Suikkala A, Leino-Kilpi H (2005). Nursing student-patient relationship: experiences of students and patients[J]. Nurse Educ Today.

