# Supplementary material for: Developing and validating the nurse-patient relationship scale (NPRS) in China
Source: BMC Nurs. 2024 Apr 22;23:255. doi: 10.1186/s12912-024-01941-w (PMC11034141; doi:10.1186/s12912-024-01941-w)
Supplement: Supplementary file 1 — Supplementary Material 1 [file 12912_2024_1941_MOESM1_ESM.docx]

**Table S1. Summary of the first round of expert consultations**

| **Original item** | **Modification** |
| --- | --- |
| I understand what it's like to be sick | I can understand and respect the feelings of patients when they are sick |
| I can't call the patient kindly | I can call the patient kindly |
| I don't like to spend time listening to patients express concerns about their illness | I'm not willing to spend time listening to patients' concerns about their condition |
| I have no patience for patients with poor expression skills | I am also patient with patients who cannot describe the disease in detail |
| Patients show bias and discrimination against my work | Patients show bias and discrimination against the nature of my work |
| The patient's address to me is rude | The patient is very rude to me |
| Patients or family members often supervise me when administering medication | When caring for a patient, the patient or the patient ’s family often supervises me |
| I think my words are easy to understand and I don't need to spend time explaining them to the patient | I think I have clearly expressed my meaning and I don’t need to spend time explaining to patients |
| In the process of communication, the patient's family members often speak excessively | During the communication process, the patient or the patient's family often express excessive emotion |

**Table S2. Item loadings on pre-defined domains: CFA Results (n=290)**

| **Factor** | Loading |
| --- | --- |
| **Nursing behavior** |  |
| I can basically solve the patient's nursing problems | 0.809 |
| When a patient has an emergency, I can correctly judge and deal with it according to the nursing standard | 0.798 |
| I can relieve the pain and stress of patients through my nursing work | 0.768 |
| I can give patients routine nursing operations in a timely manner | 0.881 |
| I have enough time and ability to give patients corresponding guidance and health education | 0.895 |
| I encourage patients to call me when they have problems | 0.779 |
| **Nurse understanding and respect for patient** |  |
| I can protect the patient's information and privacy | 0.751 |
| I can call the patient affectionately | 0.840 |
| I have no prejudice against the patients I care for | 0.821 |
| I can understand and respect the feelings of patients when they are sick | 0.885 |
| I am also patient with patients who cannot describe the disease in detail | 0.853 |
| **Patient misunderstanding and mistrust in nurse** |  |
| When caring for a patient, the patient or the patient ’s family often supervises me | 0.664 |
| Patients do not trust my explanation and health education | 0.859 |
| patients have questioned the performance of my nursing operations and professional skills | 0.854 |
| patient is very rude to me | 0.772 |
| During the communication process, the patient or the patient's family often express excessive emotion | 0.683 |
| **Communication with patient** |  |
| I think a lot of the patient's words are useless, so I will interrupt him / her soon | 0.786 |
| I think I have clearly expressed my meaning and I don’t need to spend time explaining to patients | 0.790 |
| I do not have enough energy to patiently answer questions from patients or their families | 0.836 |
| I'm not willing to spend time listening to patients' concerns about their condition | 0.518 |
| **Interaction with patient** |  |
| Before special examination or surgery, I can inform the patient of the matters needing attention in time | 0.775 |
| Maintain proper eye contact when communicating with patients | 0.929 |
| patient or family member will thank me for the care operation | 0.907 |
