# Supplementary material for: Developing and validating the nurse-patient relationship scale (NPRS) in China
Source: BMC Nurs. 2024 Apr 22;23:255. doi: 10.1186/s12912-024-01941-w (PMC11034141; doi:10.1186/s12912-024-01941-w)
Supplement: Supplementary file 2 — Supplementary Material 2 [file 12912_2024_1941_MOESM2_ESM.docx]

**Table S3. The Nurse-Patient Relationship Scale (NPRS)**

| **Nursing behavior** | **Strongly disagree** | **Disagree** | **Generally** | **Agree** | **Strongly agree** |
| --- | --- | --- | --- | --- | --- |
| I encourage patients to call me when they have problems |  |  |  |  |  |
| I can give patients routine nursing operations in a timely manner |  |  |  |  |  |
| When a patient has an emergency, I can correctly judge and deal with it according to the nursing standard |  |  |  |  |  |
| I have enough time and ability to give patients corresponding guidance and health education |  |  |  |  |  |
| I can relieve the pain and stress of patients through my nursing work |  |  |  |  |  |
| I can basically solve the patient's nursing problems |  |  |  |  |  |
| **Nurse understanding and respect for patient** |  |  |  |  |  |
| I can understand and respect the feelings of patients when they are sick |  |  |  |  |  |
| I can call the patient affectionately |  |  |  |  |  |
| I have no prejudice against the patients I care for |  |  |  |  |  |
| I can protect the patient's information and privacy |  |  |  |  |  |
| I am also patient with patients who cannot describe the disease in detail |  |  |  |  |  |
| **Patient misunderstanding and mistrust in nurse** |  |  |  |  |  |
| The patient is very rude to me |  |  |  |  |  |
| Patients have questioned the performance of my nursing operations and professional skills |  |  |  |  |  |
| When caring for a patient, the patient or the patient’s family often supervises me |  |  |  |  |  |
| Patients do not trust my explanation and health education |  |  |  |  |  |
| During the communication process, the patient or the patient's family often express excessive emotion |  |  |  |  |  |
| **Communication with patient** |  |  |  |  |  |
| I'm not willing to spend time listening to patients' concerns about their condition |  |  |  |  |  |
| I do not have enough energy to patiently answer questions from patients or their families |  |  |  |  |  |
| I think a lot of the patient's words are useless, so I will interrupt him/her soon |  |  |  |  |  |
| I think I have clearly expressed my meaning and I don’t need to spend time explaining to patients |  |  |  |  |  |
| **Interaction with patient** |  |  |  |  |  |
| Before special examination or surgery, I can inform the patient of the matters needing attention in time |  |  |  |  |  |
| Maintain proper eye contact when communicating with patients |  |  |  |  |  |
| Patient or family member will thank me for the care operation |  |  |  |  |  |
